# Author Correction: Large area optimization of meta-lens via data-free machine learning

**DOI:** 10.1038/s44172-023-00114-y

**Published:** 2023-10-03

**Authors:** Maksym Zhelyeznyakov, Johannes Fröch, Anna Wirth-Singh, Jaebum Noh, Junsuk Rho, Steve Brunton, Arka Majumdar

**Affiliations:** 1 Department of Electrical and Computer Engineering, University of Washington, Seattle, 98195 WA USA; 2 Department of Physics, University of Washington, Seattle, 98195 WA USA; 3 Department of Mechanical Engineering, Pohang University of Science and Technology (POSTECH), Pohang, 37673 Republic of Korea; 4 Department of Chemical Engineering, Pohang University of Science and Technology (POSTECH), Pohang, 37673 Republic of Korea; 5 POSCO-POSTECH-RIST Convergence Research Center for Flat Optics and Metaphotonics, Pohang University of Science and Technology (POSTECH), Pohang, 37673 Republic of Korea; 6 Department of Mechanical Engineering, University of Washington, Seattle, 98195 WA USA

**Keywords:** Metamaterials, Electrical and electronic engineering

Correction to: *Communications Engineering* 10.1038/s44172-023-00107-x, published online 21 August 2023.

In this article the affiliation ‘Department of Chemical Engineering, Pohang University of Science and Technology (POSTECH), Pohang, 37673, Republic of Korea’ for Junsuk Rho was missing. The original article has been corrected.

