# Supplementary material for: Large area optimization of meta-lens via data-free machine learning
Source: Commun Eng. 2023 Aug 21;2:60. doi: 10.1038/s44172-023-00107-x (PMC10955872; doi:10.1038/s44172-023-00107-x)
Supplement: Supplementary file 2 — Supplementary Information [file 44172_2023_107_MOESM2_ESM.pdf]

Supplementary Information

Maksym Zhelyeznyakov[1*], Johannes Fröch[1,2], Anna Wirth-Singh[2], Jaebum Noh[4], Junsuk Rho[4,5], Steve Brunton[3] and Arka Majumdar[1,2*]

[1]Department of Electrical and Computer Engineering, University of Washington, Seattle, 98195, Washington, USA.
[2]Department of Physics, University of Washington, Seattle, 98195, Washington, USA.
[3]Department of Mechanical Engineering, University of Washington, Seattle, 98195, Washington, USA.
[4]Department of Mechanical Engineering, Pohang University of Science and Technology (POSTECH), Pohang, 37673, Republic of Korea.
[5]POSCO-POSTECH-RIST Convergence Research Center for Flat Optics and Metaphotonics, Pohang University of Science and Technology (POSTECH), Pohang, 37673, Republic of Korea.

*Corresponding author(s). E-mail(s): mzhelyez@gmail.com; arka@uw.edu;
Contributing authors: jfroech@uw.edu; annaw77@uw.edu; linkle115@postech.ac.kr; jsrho@postech.ac.kr; sbrunton@uw.edu;

Supplementary Note 1

Supplementary Note 1.1 Fabrication

All devices described and discussed in the main text (forward and PINN designed) were fabricated on the same substrate. First a $\sim$ 700 nm SiN film was deposited on a quartz wafer using plasma enhanced chemical vapor deposition (SPTS Delta LPX PECVD). A thin film of a polymer resist (ZEP 520-A) and a thin film of a discharging polymer layer (DisCharge H2O) were subsequently spun onto the sample. We then used electron beam lithography (JEOL JBX-6300FS, 100 keV, 2nA) to write the various structures. After development, a short descum step (Glow Research, Autoglow, 12 s, 100 W) was used to remove remaining resist residues and subsequently a layer of 60 nm AlOx was deposited using a home-built e-beam evaporator. After overnight lift-off in warm NMP and a further plasma cleaning step in O2, we used inductively coupled reactive ion etching (Oxford Instruments PlasmaLabSystem100) with a fluorine gas chemistry to transfer the pattern from the AlOx hard mask into the underlying SiN layer. The final thickness of the etched layer indicated a pillar height of $\sim$650nm.

Supplementary Note 1.2 Experiment set up

For intensity measurements, light from a HeNe laser was transmitted through the backside of the chip and measured on the device side using a translatable microscope relay setup. In detail, the sample was mounted at a fixed position on a kinematic holder, allowing the fine adjustment for pitch and yaw, as well as the lateral position. Light was transmitted through the substrate side and would propagate entirely in air. The resulting focusing pattern was measured using a microscope setup consisting of a Nikon 100X LU Plan Fluor objective with 0.9 NA (equal or higher to the NA of the meta-optic), a tube lens (Thorlabs), and a camera (Allied Vision ProSilica GT1930), which were mounted on a programmable automated translation stage (NewPort). Frames were acquired at specific intervals of the movement, which allowed the reconstruction of the intensity vs focal distance.

Supplementary Note 1.3 Machine specifications

Ubuntu 22.04
2x Intel E5-2620 at 2.1 GHz
NVIDIA Tesla K40 12 GB Memory running CUDA 11.4
128 GB DDR3 Memory

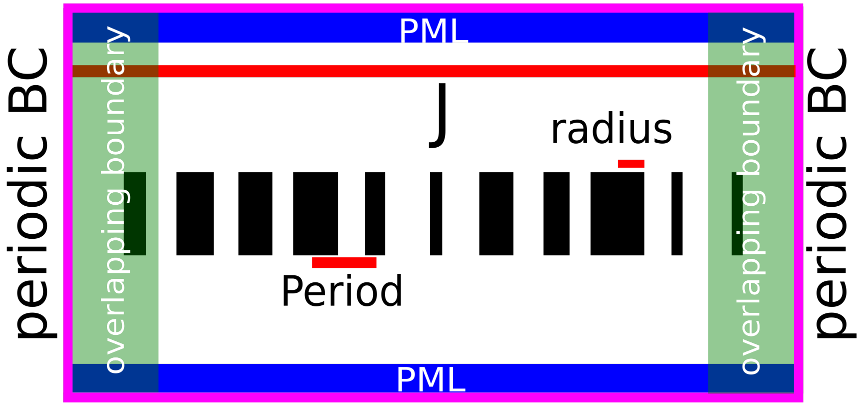

Supplementary Figure 1 Simulation problem setup

Supplementary Note 2

The neural networks are trained to predict electric field responses of distributions of dielectric scattereres, here SiN pillars, from a plane wave current source of wavelength $\lambda = 633nm$. The resolution is set to be 16 pixels per period, with each period being 0.443μm. The boundary conditions along the x-direction are set to be periodic. The boundary conditions along the y-direction are set to be 10 grid points of a perfectly matched layer (PML). The simulation domain in the x direction is $12\times$ period $= 5.316\mu$m, and $6\times$ period $= 2.658\mu$m along the y direction. When simulating field responses of large area metasurfaces, the simulation region is split up into groups of 11 periods with the outermost periods overlapping. Each region is simulated with a trained neural network as shown in Supplementary Figure 1. The field responses of the innermost 9 pillars are stitched together.

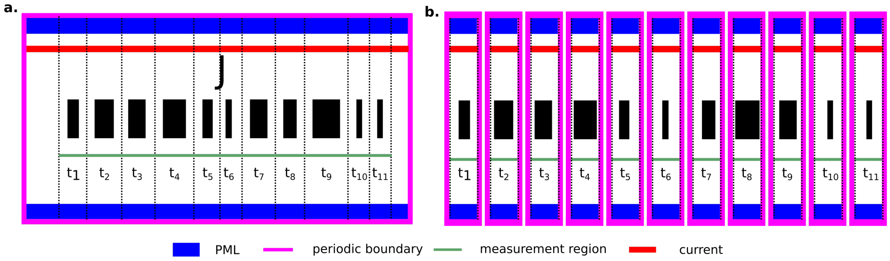

Supplementary Figure 2 Calculating pillar-wise transmission coefficients for **a.** FDFD and neural network simulations and **b.** LPA simulations.

Supplementary Note 3

Here, we summarize how we compute the average pillar-wise transmission error (i.e. main text Figure 2 **b** right hand side). For every set of 11 pillars we compute the transmission coefficients for each pillar using FDFD, PINNs, and LPA. Fig. Supplementary Figure 2 (left) shows how transmission coefficients for each batch are computed for FDFD and PINN. Fig. Supplementary Figure 2 (right) shows how transmission coefficients are computed under the LPA. For the FDFD and PINN case, we simulate the full field over the simulation region defined in the previous section. Then we measure field 6 pixels (0.13μm) away from the meta-atom. Then that field is averaged over a single period corresponding to the location of the meta-atom. This gives us 11 transmission coefficients. For the LPA, we simply simulate the 11 scatterers under periodic boundary conditions, and extract the average field the same distance away from each pillar. The mean transmission coefficient error is thus given by

$$\frac{1}{11}\sum_{i=1}^{11}|t_i^{FDFD} - t_i^{\text{approx}}|^2 \tag{1}$$

where t_i^{FDFD} is the transmission coefficient computed with FDFD and t_i^{approx} is computed by either the neural network or LPA.

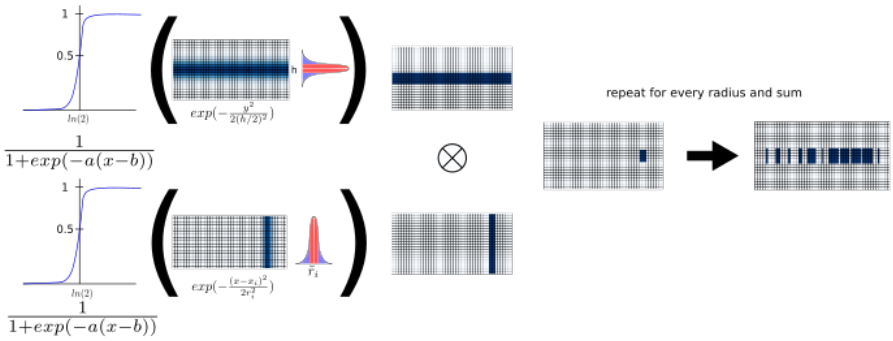

Supplementary Figure 3 transforms carried out to make a differentiable map from $r \to \epsilon$

Supplementary Note 4

To mesh a set of pillars, we first generate a grid of coordinates $x \in [-6p, 6p]$ and $y \in [-\frac{3}{2}p, \frac{3}{2}p]$, p being the pitch of the meta-optics. Since we have a fixed set of pillars, their centroid locations are given by $x_i = \frac{np}{2}$ where n is the pillar index running from -5 to 5. The first transform we define is just a shifted gaussian function on this grid:

$$T_1(r_i, x_i) = \exp\Big(-\frac{(x - x_i)^2}{2r_i^2}\Big) \tag{2}$$

Similarly, the second one defines the height:

$$T_2 = \exp\Big(-\frac{y^2}{2(h/2)^2}\Big) \tag{3}$$

The modified softmax is defined by:

$$T_3(a, b, x) = \frac{1}{1 + \exp(-a(x - b))} \tag{4}$$

a denotes the aggressiveness of the softmax function, and b is the point above which the function goes to 1, and below which the function goes to 0. Here, we chose $a = 100$ and $b = \log_e(2)$. The aggressiveness was experimentally determined, with larger values causing gradients to become too steep, and lower values causing things to not resemble pillars as much. $b = \log_e(2)$ is chosen because a gaussian function drops to $\log_e(2)$ of its max values after 1 standard deviation of its input variable, hence creating pillars of size r. Thus, 1 meshed batch of radii can be written as:

$$\sum_{i=1}^{11} T_3(T_1(r_i, x_i), 100, \log_e(2)) T_3(T_2, 100, \log_e(2)) \tag{5}$$

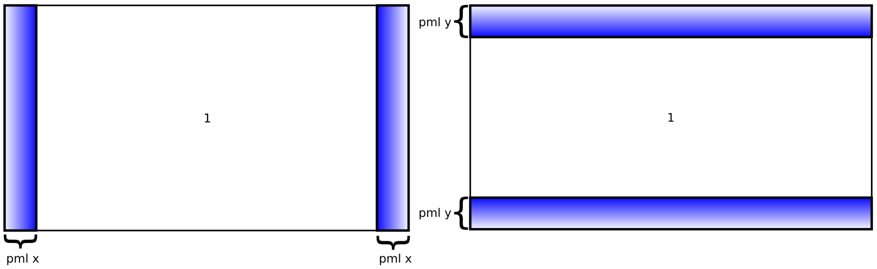

Supplementary Figure 4 Description of setup for scale matrices S_x and S_y. Left hand side is for the x derivative and right hand side is for the y derivative. PML scaling is computing on a meshed grid, then flattened and embedded in a diagonal matrix.

Supplementary Note 5

In this section we give a brief summary of formulating the FDFD linear system on a Yee grid for completeness. The full details of the method can be found elsewhere [1–4], so we only give a brief description here. The boundary conditions of Maxwell's equations are defined at $|x| \to \infty$, so perfectly matched layer (PML) boundary conditions must be implemented to truncate the simulation region to a finite size. To do this, scale matrices S_x and S_y need to be generated. They are constructed by creating scale factors

$$s_w(l) = \begin{cases} 1 - i\frac{\sigma_w(l)}{\omega_o \epsilon_0} & \text{inside } w \text{ normal pml} \\ 1 & \text{otherwise} \end{cases} \tag{6}$$

Where w is the coordinate normal (x or y), l is the distance inside the PML from the PML interface, ω_o is the operating angular frequency, and ϵ_0 is the permittivity of free space. $\sigma_w(l)$ is given by

$$\sigma_w(l) = \sigma_{w,\max}\left(\frac{l}{d}\right)^m \tag{7}$$

and $\sigma_{w,max}$ is

$$\sigma_{w,\max} = \frac{(m+1)\ln\mathrm{R}}{2\eta_0 d} \tag{8}$$

d is the thickness of the PML. η_0 is the vacuum impedance $\eta_0 = \sqrt{\mu_0/\epsilon_0}$. R is the target reflection coefficient. A good reference for PML boundaries is Shin et. al.[4]. The package we used, angler[3], uses the convention $m = 4$ and ln(R) $= -12$. Furthermore we modified the constants inside the package such that $\mu_0 = \epsilon_0 = \eta_0 = 1$. The matrices S_x and S_y are created by computing s_w on a

meshed grid, flattening it, and embedding it into a diagonal matrix:

$$S_w = \begin{bmatrix} \frac{1}{s_w^{1,1}} & 0 & 0 & \cdots & 0 \\ 0 & \frac{1}{s_w^{2,2}} & 0 & \cdots & 0 \\ 0 & 0 & \frac{1}{s_w^{3,3}} & \cdots & 0 \\ \vdots & & & \ddots & \\ 0 & 0 & 0 & \cdots & \frac{1}{s_w^{N,N}} \end{bmatrix} \tag{9}$$

The numerical derivative matrices on a yee grid, with periodic boundary conditions Δ_x and Δ_y are:

$$\mathbf{\Delta_x} = \begin{array}{r l} & \overbrace{\hphantom{-\frac{1}{dx} \quad -\frac{1}{dx} \quad \ddots \quad \frac{1}{dx}}}^{N_y} \\ (N_x-1)N_y \left\{ \vphantom{\begin{matrix} a \\ a \\ a \\ a \\ a \\ a \end{matrix}} \right. & \begin{bmatrix} -\frac{1}{dx} & & & \frac{1}{dx} & & & & \\ & -\frac{1}{dx} & & & \frac{1}{dx} & & & \\ & & \ddots & & & \ddots & & \\ & & & -\frac{1}{dx} & & & \frac{1}{dx} & \\ & & & & -\frac{1}{dx} & & & \frac{1}{dx} \\ \frac{1}{dx} & & & & & -\frac{1}{dx} & & \\ & \ddots & & & & & \ddots & \\ & & \frac{1}{dx} & & & & & -\frac{1}{dx} \end{bmatrix} \end{array} \tag{10}$$

$$\mathbf{\Delta_y} = \begin{array}{r l} & \overbrace{\hphantom{-\frac{1}{dy} \quad \frac{1}{dy} \quad \frac{1}{dy} \quad \ddots}}^{N_y} \\ N_y \left\{ \vphantom{\begin{matrix} a \\ a \\ a \\ a \end{matrix}} \right. & \begin{bmatrix} -\frac{1}{dy} & \frac{1}{dy} & & & & & & & & \\ & -\frac{1}{dy} & \frac{1}{dy} & & & & & & & \\ & & \ddots & \ddots & & & & & & \\ \frac{1}{dy} & & & -\frac{1}{dy} & 0 & & & & & \\ & & & & -\frac{1}{dy} & \frac{1}{dy} & & & & \\ & & & & & -\frac{1}{dy} & \frac{1}{dy} & & & \\ & & & & & & & \ddots & \ddots & \\ & & & & & & \frac{1}{dy} & & & -\frac{1}{dy} \end{bmatrix} \end{array} \tag{11}$$

here N_x and N_y are the grid sizes in the x and y directions and dx and dy are the grid spacings. The derivative matrices D_x^e, D_y^e, D_x^h, and D_y^h can then be constructed as:

$$D_x^e = S_x \Delta_x \tag{12}$$

$$D_y^e = S_y \Delta_y \tag{13}$$

$$D_x^h = S_x(-\Delta_x^\dagger) \tag{14}$$

$$D_y^h = S_y(-\Delta_y^\dagger) \tag{15}$$

where the † operator is the Hermitian transpose.

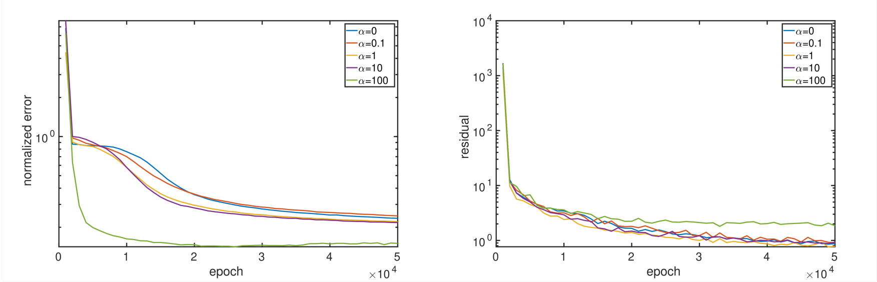

Supplementary Figure 5 Comparison of neural network performance for different values of α in the data loss term. **Left:** epoch vs normalized error given by eq. 19 and **right:** the residual. Both both plots are the done on the test data set.

Supplementary Note 6

In this section we describe the effect of adding data to the PINN loss function. Given a PINN loss:

$$f(\varepsilon;\ \theta) = \left|\left|[D_x^h D_x^e + D_y^h D_x^e + \omega_o^2 \varepsilon] NN(\varepsilon;\ \theta) + i\omega_o J\right|\right|_1 \tag{16}$$

and a data loss

$$g(\varepsilon;\ \theta) = \left|\left|E_{FDFD} - E_{NN}(\varepsilon;\ \theta)\right|\right|_1 \tag{17}$$

we can form a total loss function:

$$h(\varepsilon;\ \theta) = f(\varepsilon;\ \theta) + g(\varepsilon;\ \theta) \tag{18}$$

Where ε is the input dielectric distribution, θ are the trainable parameters of the neural network, and α is the "strength" of the data term. Fig. Supplementary Figure 5 shows the test loss vs epoch of neural networks trained with various α parameters. We generated a dataset of 10000 fields by directly simulating our problem with random pillar arrangements using angler [3]. The training was done on 9900 fields, and the test was done on the remaining 100. The left hand side of Fig. Supplementary Figure 5 shows the accuracy of the neural network given by the 2 norm relative error between the fields simulated by FDFD and the neural net:

$$\frac{||E_{FDFD} - NN||_2^2}{||E_{FDFD}||_2^2} \tag{19}$$

on the test data set. The right hand side shows the same information except for the residual given by 16. We note that, while some data parameter α may marginally improve how well the neural network predicts a field, for values of α where this improvement becomes significant, the physics informed loss takes

a penalty. We argue that having a lower physics informed loss is a much better scenario for optimization problems because it means the fields satisfy the PDE better, and thus more accurately represent the predicted physical quantity. Furthermore, the improvement from including a data term is marginal, and increases the complexity of neural network training, that it is not worth adding.

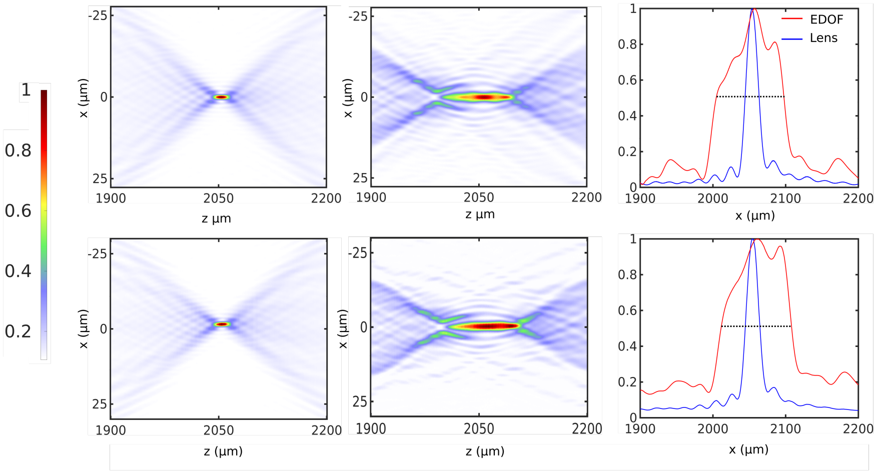

Supplementary Figure 6 EDOF lens inverse design. **a. - c.** represent theoretical results, and **d. - f.** are experimentally measured results. **a., d.** are forward designed lenses with focal length 2.05 mm. **b.,e.** are the optimized EDOF lenses. **c., f.** are slices along the z axis with $x = 0\mu$m. The red lines are the EDOF and the blue lines are the lens. The black line is plotted at the full width half maxima of the EDOF lens, which is how we define the depth of focus[5]. The theoretical and experiment depth of focus for the forward designed lens is 20μm. The EDOF lens has a theoretical depth of focus of 93μm and an experimentally measured depth of focus of 97μm

.

Supplementary Note 7

We also designed an EDOF lens through a standard max-min objective approach, where we computed the intensity of the field produced by our lens at discrete equidistantly spaced points along a focal line, on an interval between two different focal lengths:

$$\begin{gathered} f = \{f_1, f_2, ..., f_{10}\} \\ f_i = E^\dagger(0, z_i)E(0, z_i) \\ \max_r \min_{f_i} f \end{gathered} \tag{20}$$

We then used forward design to generate a lens with focal length 2050μm and a diameter of 1000μm, and optimized an EDOF lens to extend its focus between 2000 and 2100 microns. Fig. Supplementary Figure 6 shows a summary of the results for the inverse designed EDOF lens. To characterize the performance of the lens, we define the depth of focus as the point where the distance at which the focal spot intensity reaches $1/2$ of its original value, see Fig. Supplementary Figure 6 c and f. The EDOF device has a theoretically predicted depth of focus of 93μm and an experimentally measured depth of focus of 97μm. The depth of focus of the forward designed lens is $\sim 20\mu$m measured both in experiment and theory.

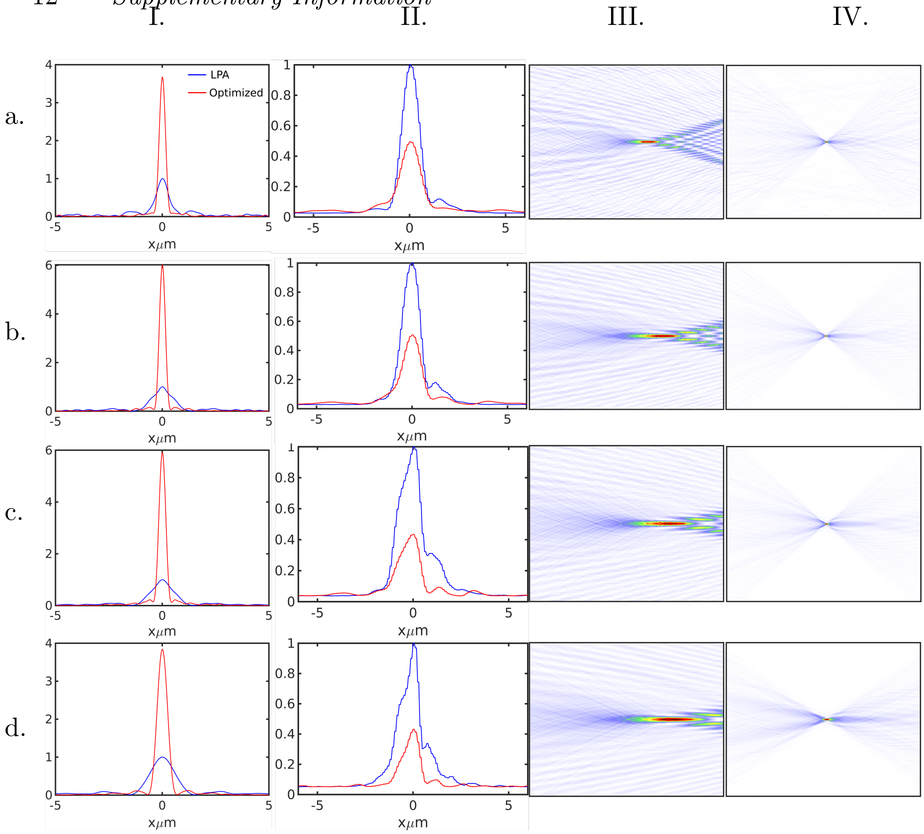

Supplementary Figure 7 **I** Theoretically predicted intesnities at the focal spot. **II.** Experimentally measured intensities at the focal spot. **III.** Theoretically predicted focal spot for forward design **IV** and inverse design. **a.** 200 μm focal length. **b.** 500 μm **c.** 750 μm **d.** 1000μm.

Supplementary Note 8

We want to emphasize the importance of experimentally verifying inverse design methods. Initially we designed our inverse design method by extracting derivative matrices from angler [3], and comparing our results to the FDFD results from the same package in order to make sure our results were consistent and accurate. Fig. Supplementary Figure 7 summarizes our results. We note there there is a large discrepancy between the theory and experiment here. This is due to the fact that angler and our first iteration of our inverse design methodology was predicted the complex conjugate of the electric field instead of the electric field, leading to angular spectrum propagation to propagate the fields in the opposite direction. We would never have realized this error if we did not conduct experimental testing of our devices. We encourage more inverse design papers in the future to manufacture and test their inverse design methods in order to produce reliable and accurate designs.

	PINN				FDFD	
	Batch Size 1	Batch Size 10	Batch Size 20	Batch size 40	Overlapping BCs	Full Simulation
average time per chunk (s)	0.006	0.004	0.004	0.005	0.021	
total sim time (s)	1.17	1.04	1.17	1.13	5.29	11.43
Ram Usage (GB)	5.29	5.29	5.29	5.29	1.77	36.7
GPU Memory (GB)	1.62	2.88	4.58	7.17		

Supplementary Table 1 Resource and speed comparisons between the PINN approach and the FDFD approach for the forward simulation of a 1mm lens. The PINN approach is about 5x faster than the FDFD approach when overlapping boundary conditions are used and 10x faster when we don't use overlapping boundary conditions. Furthermore, the overlapping boundary method is more memory efficient, and thus useful for running inverse design on machines with low RAM.

Supplementary Note 9

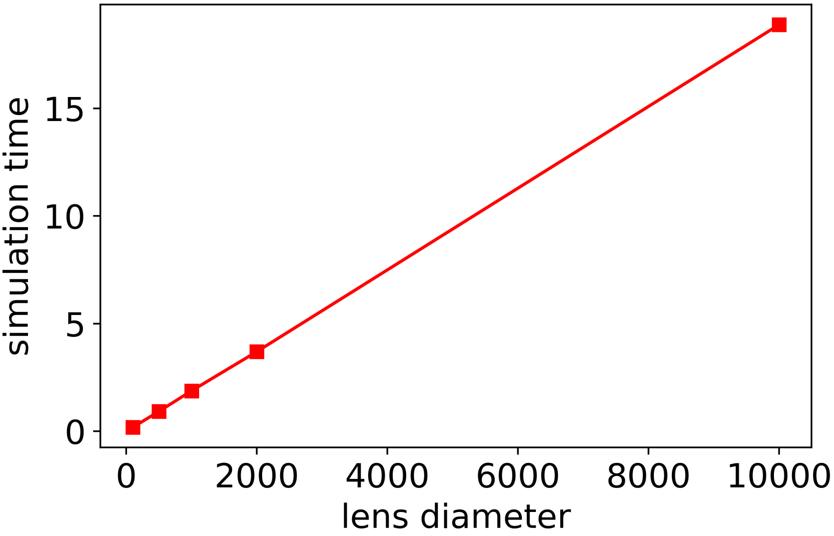

Supplementary Figure 8 Comparison of neural network simulation time vs lens diameter. Data is taken for lens diameters of 100, 500, 1000, 2000, and 10000 μm lens diameters. Simulation time increases linearly with lens diameter.

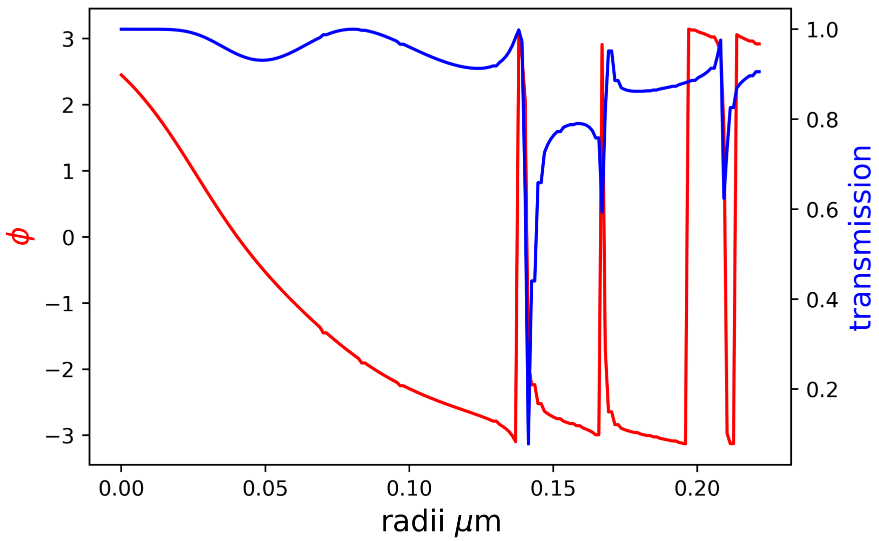

Supplementary Figure 9 Phase and amplitude transmission for the pillars with height $h = 0.6$, refractive index $n = 2$, and periodicity 0.443μm at operating wavelength $\lambda = 0.633\mu$m

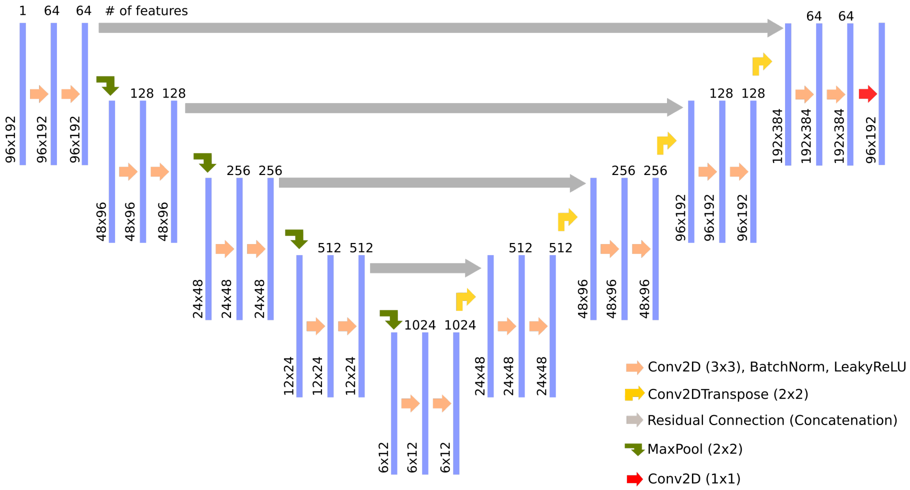

Supplementary Figure 10 Detailed neural network architecture

Supplementary References

[1] Rumpf, R.: Electromagnetic and Photonic Simulation for the Beginner: Finite-difference Frequency-domain in MATLAB (R). Artech House, Norwood, MA (2022)

[2] Rumpf, R.C., Garcia, C.R., Berry, E.A., Barton, J.H.: Finite-difference frequency-domain algorithm for modeling electromagnetic scattering from general anisotropic objects. Progress in Electromagnetic Research B **61**, 55–67 (2014)

[3] Hughes, T.W., Minkov, M., Williamson, I.A.D., Fan, S.: Adjoint method and inverse design for nonlinear nanophotonic devices. ACS Photonics **5**(12), 4781–4787 (2018). https://doi.org/10.1021/acsphotonics.8b01522

[4] Shin, W., Fan, S.: Choice of the perfectly matched layer boundary condition for frequency-domain maxwell's equations solvers. Journal of Computational Physics **231**(8), 3406–3431 (2012). https://doi.org/10.1016/j.jcp.2012.01.013

[5] Bayati, E., Pestourie, R., Colburn, S., Lin, Z., Johnson, S.G., Majumdar, A.: Inverse designed metalenses with extended depth of focus. ACS Photonics **7**(4), 873–878 (2020). https://doi.org/10.1021/acsphotonics.9b01703
